# Performance Optimization of a Developed Near-Infrared Spectrometer Using Calibration Transfer with a Variety of Transfer Samples for Geographical Origin Identification of Coffee Beans

**DOI:** 10.3390/molecules27238208

**Published:** 2022-11-25

**Authors:** Nutthatida Phuangsaijai, Parichat Theanjumpol, Sila Kittiwachana

**Affiliations:** 1 Department of Chemistry, Faculty of Science, Chiang Mai University, Chiang Mai 50200, Thailand; 2 Postharvest Technology Research Center, Faculty of Agriculture, Chiang Mai University, Chiang Mai 50200, Thailand; 3 Postharvest Technology Innovation Center, Ministry of Higher Education, Science, Research and Innovation, Bangkok 10400, Thailand

**Keywords:** near-infrared (NIR) spectrometer, geographical origin, calibration transfer, coffee, supervised self-organizing map (SSOM), classification

## Abstract

This research aimed to improve the classification performance of a developed near-infrared (NIR) spectrometer when applied to the geographical origin identification of coffee bean samples. The modification was based on the utilization of a collection of spectral databases from several different agricultural samples, including corn, red beans, mung beans, black beans, soybeans, green and roasted coffee, adzuki beans, and paddy and white rice. These databases were established using a reference NIR instrument and the piecewise direct standardization (PDS) calibration transfer method. To evaluate the suitability of the transfer samples, the Davies–Bouldin index (DBI) was calculated. The outcomes that resulted in low DBI values were likely to produce better classification rates. The classification of coffee origins was based on the use of a supervised self-organizing map (SSOM). Without the spectral modification, SSOM classification using the developed NIR instrument resulted in predictive ability (% PA), model stability (% MS), and correctly classified instances (% CC) values of 61%, 58%, and 64%, respectively. After the transformation process was completed with the corn, red bean, mung bean, white rice, and green coffee NIR spectral data, the predictive performance of the SSOM models was found to have improved (67–79% CC). The best classification performance was observed with the use of corn, producing improved % PA, % MS, and % CC values at 71%, 67%, and 79%, respectively.

## 1. Introduction

Coffee is among the most popular beverages consumed by people from all over the world. Coffee can be grown in many areas, especially in the equatorial region. However, it has been reported that the conditions of the growing environment can affect the chemical composition of coffee beans and consequently influence the characteristic quality of the coffee products [1]. Up to the present, several analytical techniques have been utilized for investigating the geographical origins of coffee samples, such as nuclear magnetic resonance (NMR), X-ray fluorescence (XRF), high-performance liquid chromatography (HPLC), and gas chromatography-mass spectrometry (GC-MS) [2]. Nevertheless, these analysis techniques require complicated sample preparation. In addition, the analysis cost can be high, especially for a large number of samples. Near-infrared (NIR) spectrometry has been among the most promising analytical tools for a variety of agricultural products [3]. This detection method inspects the interaction between samples and electromagnetic light in a region between 800–2500 nm, relating to the overtone and combination bands of fundamental molecular vibrations from the infrared (IR) region [4,5]. NIR detection, regarded as reagentless detection, could be suitable for this qualitative analysis task, offering the advantage that NIR spectra could be rapidly and directly acquired from coffee samples without the need for sample preparation.

However, the cost of a commercial benchtop NIR spectrometer can be relatively expensive when compared to other analytical instruments that employ similar detection methodology, such as ultraviolet (UV) and visible (VIS) spectrometers. This could be among the main drawbacks that make the practical use of NIR detection rather limited, especially for agricultural applications wherein the products are not expensive. Recently, various low-cost NIR spectrometers have been developed [6,7,8]. For example, a fiber-optic NIR spectrometer based on micro-electromechanical systems (MEMS) detection was developed for monitoring total acids (TA) and total polyphenol content (TPC) in the fermentation process of mulberry vinegar [9]. The comparative use of an NIR system utilizing MEMS technology for the spectrochemical detector has been reported [10]. The developmental process included the use of miniature portable instruments, offering opportunities for on-site analysis outside the laboratory [11]. However, the major trade-off was that the performance of the low-cost or homemade system could not compete with the well-established and commercial benchtop NIR spectrometer. For instance, most of the homemade NIR detectors were designed to capture only a fraction of the light spectrum. A conventional Hamamatsu InGaAs image sensor (Hamamatsu Photonics K.K., Hamamatsu City, Japan) recorded NIR absorbance in the region of 750–1050 nm when compared to a commercial benchtop NIR spectrometer (NIRSystem 6500, Foss NIR Systems, Silver Spring, MD, USA) with recorded values in the region of 400–2500 nm. In addition, certain instabilities emerged due to environmental conditions, such as the interference of ambient light and changing detector temperatures. These conditions could also weaken the reliability of the fabricated instruments.

Theoretically, more useful variations can be extracted from a commercial benchtop NIR instrument, resulting in a better degree of predictive performance when compared to those obtained from homemade or low-cost NIR instruments. Therefore, the predictive ability of the counterpart NIR systems could be improved by incorporating the essential information found in the reference system with greater spectral detection ability. Calibration transfer involves a group of chemometric methods that mathematically create a connection between the data obtained from different detection instruments. These are often referred to as master and slave instruments [12]. Piecewise direct standardization (PDS) is one of the powerful calibration methods of transfer that can be used to create a correlation model between the two instruments. The resulting correlation information is then used to establish standardization among the spectral differences [13]. PDS can be adopted for both qualitative [14] and quantitative analyses [15] to adjust the slave spectra to be fitted with the model established from the reference or master instrument. After being adjusted, the data obtained from different instruments can be exchanged without the need to recalibrate the prediction models.

Ideally, the NIR spectra of the identical samples—the so-called “transfer samples”—should be recorded from both the master and the slave instruments to establish an accurate calibration transfer model [16]. In addition, previous studies have reported that the transfer samples should be of the same makeup as those of the test samples. For example, for the determination of some quality-related parameters in apples, spectral data from the apple should be used to construct the applicable standardization model [17]. This could be among the limitations that have emerged in adapting calibration transfer methods, since an additional set of the specific transfer samples is required every time new samples are introduced for spectral detection.

This research aimed to classify green coffee bean samples based on their growing origins with the developed NIR detection, using artificial neural network classification. The predictive performance of the developed NIR instrument was improved based on spectral transformation using the PDS calibration transfer method. The effects of different types of transfer samples on the instrumental calibration process were investigated. The PDS transformation using green coffee beans and the classification based on the NIR reference instrument was performed for comparison and for demonstration of the improvement achieved by the developed method. This development allowed the homemade NIR system to provide robust and accurate prediction results based on utilization of the existing NIR spectral databases acquired from the reference instruments.

## 2. Results and Discussion

### 2.1. Exploratory Data Analyses of NIR Spectra

**Figure 1:**
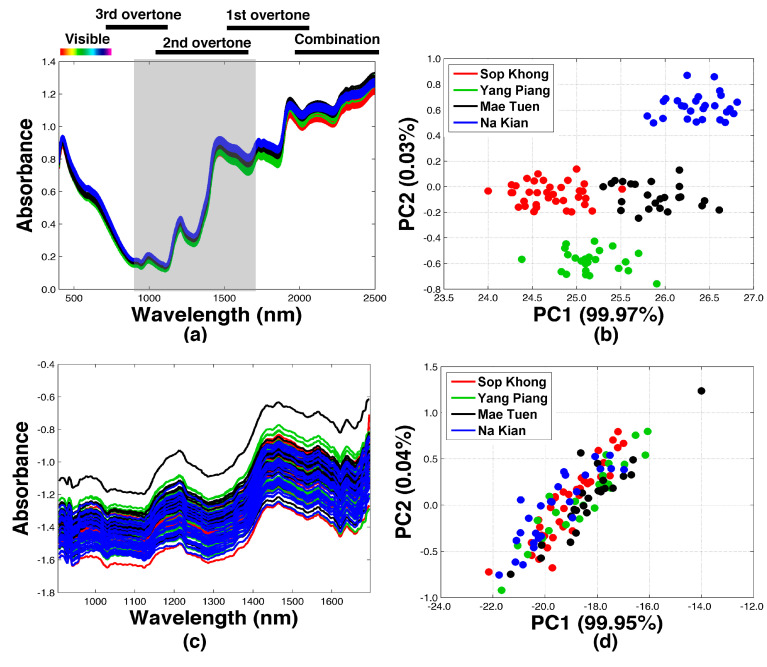
NIR spectra of the coffee samples obtained from: (**a**) NIR Foss 6500 and (**c**) the developed NIR spectrometer. PCA score plots of the NIR spectra obtained from (**b**) NIR Foss 6500 and (**d**) the developed NIR spectrometer.

Figure 1 presents the NIR spectra of the studied coffee samples. The NIR spectra established from the reference instrument are presented in Figure 1a and the corresponding principal component analysis (PCA) analysis is presented in Figure 1b. The PCA score plot revealed four different clusters of the samples, implying that the reference instrument could successfully capture the differences among coffee beans obtained from different geographical origins. In contrast, the NIR spectra established from the developed system in Figure 1c were prone to having more deviations. In addition, the samples could not be separated and were clustered into a single group on the PCA space on Figure 1d. These results demonstrated that the homemade instrument presented an inferior degree of NIR spectral quality for this study case.

**Figure 2:**
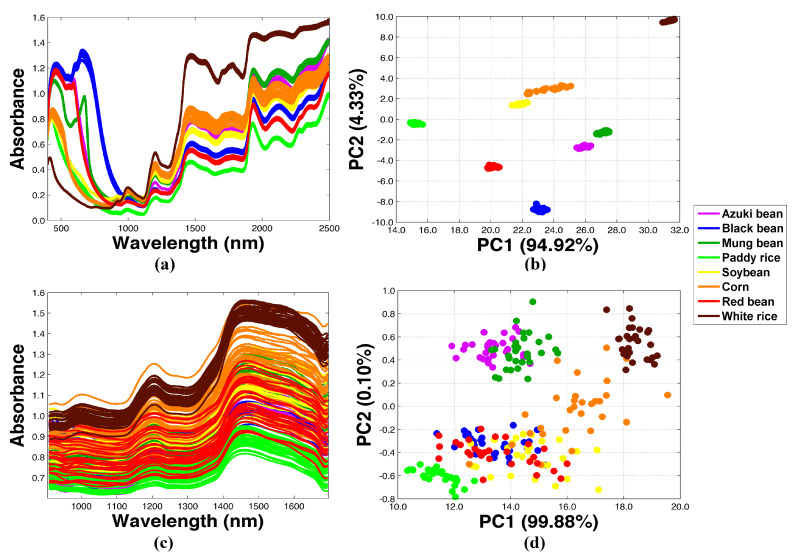
NIR spectra (**a**,**c**) and corresponding PCA (**b**,**d**) of the transfer samples. Data were established from both the reference and the homemade instruments, respectively.

The NIR spectra recorded from the transfer samples using the reference and the homemade systems are presented in Figure 2a and Figure 2c, respectively. The corresponding PCA analyses of the NIR datasets are visualized in Figure 2b,d. The NIR spectra of green coffee beans from the MT plantation were also included. The chemical and physical variations of each agricultural product were different, and therefore were reflected in the samples organized on the PCA score plots, implying that both NIR spectrometers could be used to identify differentiations among the agricultural samples. It should be noted that the coffee bean roasting process caused intense chemical changes and resulted in significant variations in the NIR spectra and the PCA score. Therefore, the PCA results were not included for clearer comparison among the other agricultural products.

### 2.2. Transformation of NIR Spectra Using Different Agricultural Samples

**Figure 3:**
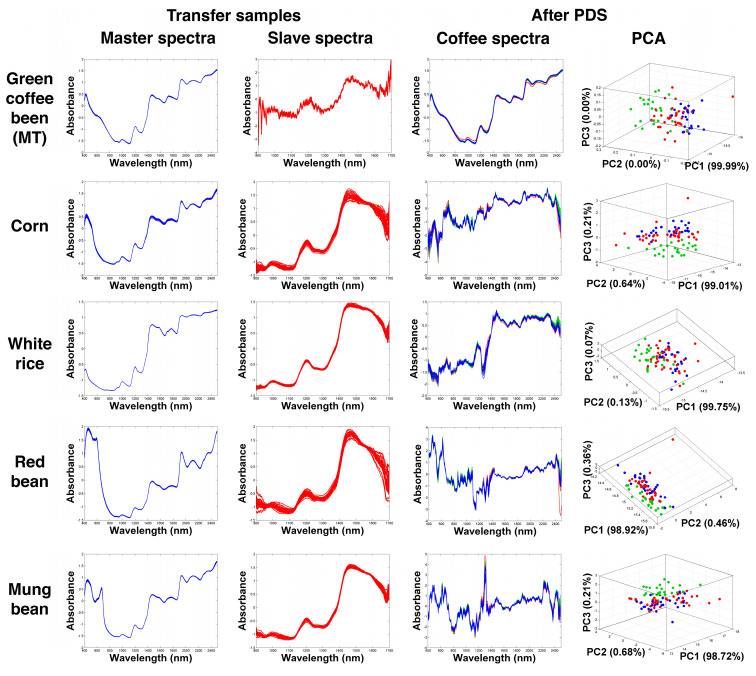
NIR spectra of green coffee beans after PDS transformation using green coffee beans (MT), corn, white rice, red beans, and mung beans, and the corresponding PCA score plots compared with the NIR spectra of the transfer samples.

**Figure 4:**
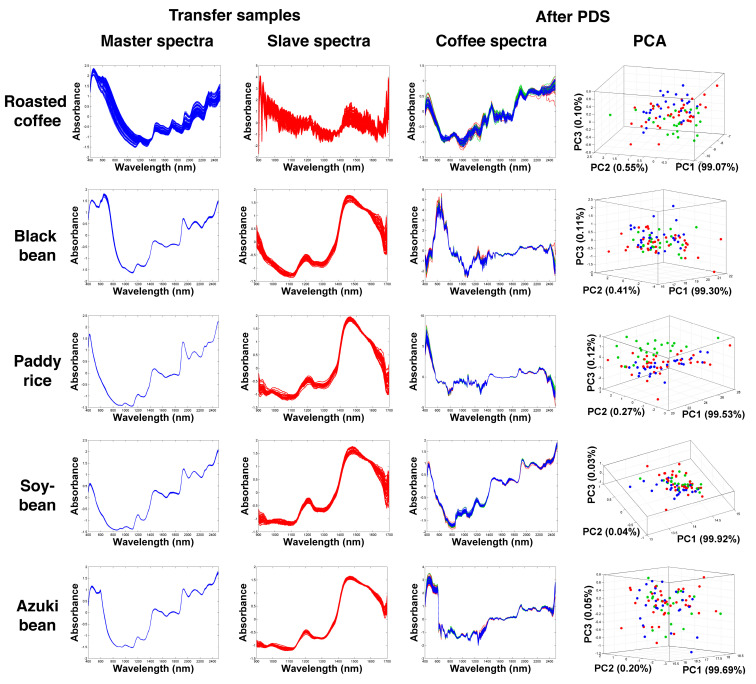
NIR spectra of green coffee beans after the PDS transformation using roasted coffee, black beans, paddy rice, soybeans, and azuki beans and the corresponding PCA score plots compared with the NIR spectra of the transfer samples.

Figure 3 and Figure 4 demonstrate the NIR spectra after the PDS process was completed using different transfer samples. The spectra of each agricultural transfer sample established from both NIR systems were compared. In addition, the spectral data acquired from the homemade instrument after the PDS process were presented together with the corresponding PCA modelling. In Figure 3, using the coffee obtained from the MT plantation as the transfer sample, it was observed that the shape of the standardized spectra significantly changed and became very similar to that of the transfer samples that were recorded using the NIR 6500 system. It should be noted here that the amount of the absorption data after the transformation was limited by the number of the absorption parameters of the slave instrument, which in this case was 255 parameters.

The PDS transformation subsequently created local multivariate models. The created local models assumed that the spectral information at a certain wavelength of the slave instrument was contained in a small spectral region of the neighboring wavelengths of the master instrument. However, the low-cost NIR instrument only captured the short-wave spectral information between 900–1700 nm, which was considered the second overtone NIR region (Figure 1a). The absorption values of the fundamental frequencies or overtone bands of the same chemical functional groups were also present in the other regions (combination of the first and the third regions) [18]. Therefore, by using the MT samples to establish the transfer samples, the reconstruction of the spectra in the other NIR regions could be enabled based on the correlation information in the established transfer matrix (***F***). In this case, after PDS transformation, an improvement could be clearly noticed from the PCA score plot. Although all samples were still organized into a single cluster, the regions of the coffee samples obtained from the different plantation areas became clearer when compared to the PCA results in Figure 1d.

PCA is an unsupervised learning method and directly reflects the differences of spectra. There were differences among the spectra recorded by the reference spectrometer, which led to the sample cluster on the PCA score plots. For the spectra recorded by the homemade spectrometer, the differences did not seem significant. After spectra transfer process, the unsupervised learning method PCA was changed to a supervised learning method, improving the performance of the instrument. On the score plot, the green coffee beans obtained from YP (red circles) could be separated from those that were obtained from NK (blue circles).

In considering the use of different agricultural products as transfer samples, in particular corn, red beans, and white rice as shown in Figure 3, the transferred NIR spectra dramatically changed in terms of shape. Unlike when the MT transfer samples were used, the resulting figures of the transferred spectra were significantly different from the master spectra. Changes in the spectral shapes were quite normal and could be the same as when common pretreatment methods were used, such as scaling, centering, and normalization. Still, the coffee samples obtained from different plantations could be observed on the PCA spaces, implying a degree of improvement after the transformation process. However, when using paddy rice, soy, and adzuki beans as shown in Figure 4, the spectral reconstructions did not result in significant improvement of the sample organization, and most of the samples were placed on the overlapping areas of the PCA spaces.

The developed method aimed to classify the geographical origins of the coffee bean samples. It should be noted here that the transfer samples were chosen based on two important criteria. Firstly, the transfer samples should share some common properties with the test samples, such as moisture and graininess. Secondly, their main compositions should be similar. For example, carbohydrates, proteins, and fats were among the main components in the grain samples. It was not required that all the grain samples contain caffeine as in the coffee beans, because the NIR detection mainly captured the variation from the major components in the samples.

### 2.3. Comparison of Classification Performance

#### 2.3.1. Classification Results Using Uncalibrated Spectra

Table 1 presents the classification results of the coffee bean samples using SSOM. Without PDS transformation, the reference instrument could successfully provide the best classification performance in obtaining the % PA, % MS, and % CC values of 98%, 96%, and 100%, respectively. Higher values of % PA, % MS, and % CC (close to 100%) indicated greater degrees of accuracy, stability, and performance of the classification models [19]. In contrast, the homemade instrument resulted in significantly lower classification performance, with % PA, % MS, and % CC values in the test mode at 61%, 58%, and 64%, respectively. These classification results were as expected and corresponded to the poor organizational structure on the PCA space presented in Figure 1d. Although the performance of the fabricated NIR instrument was relatively lower, it highlighted the potential use of the developed instrument for agricultural applications.

#### 2.3.2. Classification Results Using Transformed Spectra

After the PDS transformation used different transfer samples, the classification results were changed. Using MT green coffee as the transfer sample, the classification accuracy increased to 78% CC. This outcome suggested that transfer samples with the same characteristics as the test or unknown samples could enhance the classification performance of the homemade instrument, thereby demonstrating that the calibration transfer method could be used to improve the classification results. Interestingly, using corn, red beans, and white rice as the transfer samples, the classification performance in terms of the % CC was also clearly improved (75–79% CC), where the best classification value was obtained from SSOM using corn as the transfer sample (79% CC). However, the use of black beans and roasted coffee did not significantly improve the predictive performance when compared with the predictions that were based on the non-transformed spectra. In contrast, adzuki beans, paddy rice, and soybeans unsuccessfully offered lower % CC values (52–59%).

#### 2.3.3. Evaluation of Improvement after Spectral Transformation

To monitor improvement after PDS transformation, DBI values of the transformed spectral data using different transfer samples were calculated and are listed in Table 1. Ideally, the DBI values were calculated as the ratio between the intra-cluster distances among the samples and the distance between the cluster centroids. These values were reported as relative constants where smaller values of DBI indicated a better degree of clustering quality. In this study case, the smallest DBI value of 0.39 was obtained from the classification data using the NIR spectra data established from the reference instrument, which resulted in the best classification result of 100% CC. At the same time, the NIR spectra established from the homemade instrument without the transformation reported a DBI value of 5.75, which resulted in a poorer classification result of 64% CC.

**Figure 5:**
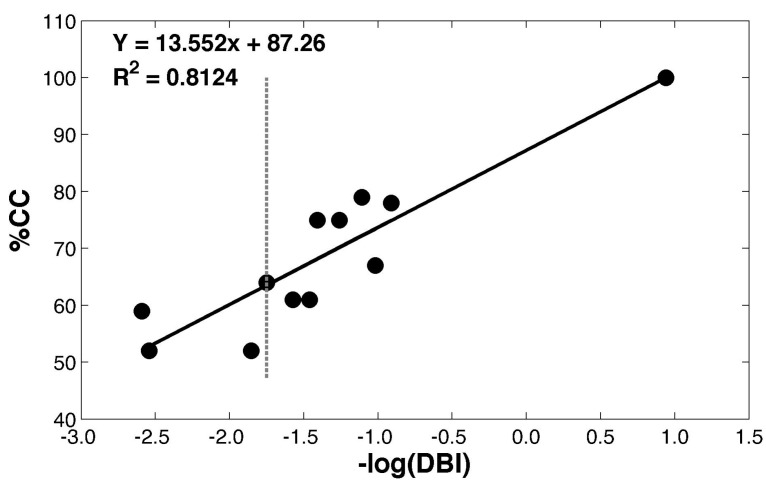
Correlation between the −log(DBI) and the % CC values. The vertical dotted line indicates the DBI values of the predictive results from the classification model using the homemade NIR instrument, without transformation.

The transfer samples resulted in increased % CC values of the MT green coffee beans, corn, red beans, mung beans, and white rice by providing DBI values ranging from 2.49 to 4.09, whereas adzuki beans, paddy rice, and soybeans exhibited an increase in DBI values ranking from 6.38 to 13.36. Figure 5 shows a correlation plot between the −log(DBI) values and the classification performance in terms of % CC based on SSOM classifications. A high R^2^ value of 0.816 indicated a strong relationship between the −log(DBI) values and the % CC predictive performance. This outcome implied that DBI could be used to evaluate the degree of improvement of the NIR spectral data after PDS transformation.

In this research, a methodology to improve the classification performance of a homemade NIR spectrometer aiming to identify the geographical origins of coffee beans was described. The development was based on the learning and transformation of essential information found in the reference system with greater spectral detection ability, called the transfer samples. In addition, a variety of agricultural samples was employed to serve as the transfer samples in the transformation process. This research has demonstrated that it was not required that transfer samples should be the same type as the test samples. Different types of transfer samples could be used for the transformation process and improve the classification ability.

In ideal work conditions, it would be recommended that large numbers of different transfer samples be tested and compared by the calculation of the DBI values. A strong relationship between the −log(DBI) values and the % CC predictive performance could be observed. Therefore, it was possible to forecast the degree of improvement of the NIR spectral data after PDS transformation when the different transfer samples were used. The transformed NIR spectra with low DBI values tended to offer improved classification results. However, it should be noted that the developed method could not point out the reason why transformation based on the selected samples could result in optimal predictive results. The suitability of the transfer samples should be re-evaluated and compared if new transfer samples are introduced to the database.

## 3. Materials and Methods

### 3.1. Green Coffee Bean Samples

Green coffee beans were collected from four different subdistricts located in Chiang Mai, the largest province in the northern part of Thailand, in 2020, including Sop Khong (SK), Na Kian (NK), Yang Piang (YP), and Mae Tuen (MT). Although these plantation areas are in the same province, their environmental conditions are rather different due to their elevation above sea level (1107, 1212, 936, and 441 m for SK, NK, YP, and MT, respectively). Characteristic details of the coffee samples have been presented in Table 2. The samples (SK, NK, and YP) were categorized into three class memberships according to their geographical origins, whereas the MT samples were used as the reference transfer samples.

### 3.2. Near-Infrared Instruments (NIRs)

In this research, two NIR spectrometers were used as the reference and homemade spectrometers. For the reference NIR system, a Foss NIRSystem 6500 (Foss NIR Systems, Silver Spring, MD, USA) benchtop instrument was used. The detection of the reference instrument was in the region of 400–2500 nm at 2 nm sampling intervals using an NIR reflectance transportation module in a 25 °C temperature-controlled room. This detection resulted in a total of 1050 absorption parameters. For 100 g of each sample, the spectral data from 64 detection points were averaged to provide a mean spectrum.

**Figure 6:**
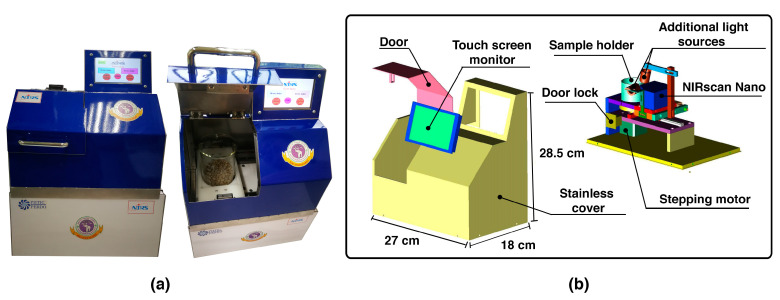
Homemade NIR spectrometer; (**a**) front side view and (**b**) diagram showing the setting of the NIR sensor.

For the low-cost NIR system, a homemade NIR spectrometer that had been described in previously published literature [20] was used. This homemade NIR spectrometer was fabricated using a single-element NIR detector (DLP NIRscan Nano, Texas instruments, Dallas, TX, USA) to measure the NIR light from 900 nm to 1700 nm in a reflectance mode. A total of 255 absorption parameters were recorded. A commercial quartz beaker 400 mL (PYREX, Germany) was used as a sample cell, where seven spectra were recorded from seven different points to provide a mean spectrum value. An LED touch screen was used to control and operate the measurement process; a homemade control program was written based on the Raspberry PI platform. In addition, a USB connector was used to connect the system to a personal computer for additional data processing via the MATLAB program (MATLAB V10.0, The MathWorks Inc., Natick, MA, USA). The details of the fabricated NIR spectrometer are presented in Figure 6. Prior to NIR analyses using both instruments, the coffee samples were stored in a temperature-controlled room at 25 °C for at least 6 h.

The performance of these two spectrometers has been previously compared and reported. Generally, the detection performance of the homemade NIR spectrometer could not compete with that of the commercial NIR spectrometer [20,21]. However, the homemade NIR system was associated with an easier process of implementation. Furthermore, the cost of the homemade NIR instrument was much lower, thus representing a very attractive alternative for determining agricultural quality.

### 3.3. Chemometric Analysis

#### 3.3.1. Piecewise Direct Standardization (PDS)

Calibration transfer (CT) methods were used to investigate the relationship between the spectral data obtained from the reference (master) and the other (slave) instruments. This relationship information was then used to adjust for the differences between the variations recorded from both instruments. Piecewise direct standardization (PDS) involves the improvement of a conventional calibration transfer algorithm called direct standardization (DS), where the data are segmented into small windows so that the spectral adjustment of the slave instrument can be based on the local characteristics rather than on the entire spectrum of the reference [22].

In this research, the NIR spectra established from the reference and the low-cost spectrometers were defined as ***X*_MS_** and ***X*_SL_**, respectively. After the PDS process, the relationship information was expressed in terms of a transformation matrix (***F***). In this research, partial least squares (PLS) regression was used to evaluate this relationship information [23]. A total of 10 different sets of the transfer samples resulted in 10 different transformation matrices where the classification performances were compared. The PDS calculation has been described in previously published literature [24] and the parameters were set according to this report [25].

##### Transfer Samples

To investigate the transfer ability using different types of samples, different agricultural products, including corn, red beans, mung beans, black beans, soybeans, green and roasted coffee, adzuki beans, and paddy and white rice were adopted as the transfer samples. The developed method aimed to classify the geographical origins of the green coffee bean samples. Therefore, the transfer samples used in the current study were restricted to grain products. The number of transfer samples used could affect the modelling ability of the standardized spectra [22]; however, the number of these transfer samples were fixed to 30 samples for the purposes of comparison. All the samples were purchased from local markets in Chiang Mai Province. NIR spectra were recorded from both NIR spectrometers and used as the transfer spectral data.

#### 3.3.2. Self-Organizing Map (SOM) for Classification

The Kohonen map, or self-organizing map (SOM), is among the most well-known artificial neural networks that can be used to project data from a high-dimensional space onto a low-dimensional array of neurons (or map units) [26]. Supervised SOM (SSOM) is an extension of SOM, where the information of class membership data is provided during the model training process [27]. After the training process, the class prediction of unknown samples can be achieved by identifying the class membership of the map unit when it is placed on the trained map, or of the best matching unit (BMU) [28]. Since SSOM constructed the models based on characteristic variations in the training samples, no mathematic functions were needed to describe the data variation, and so it was considered a nonlinear classifier. Several studies have reported that for NIR spectral analysis, nonlinear predictions could provide optimal predictive results when compared to conventional linear methods [29]. The calculation of an SSOM was used for the purposes of classification, while the parameter sets used have been described in a previous study [27]. Appropriate spectral pretreatments could provide optimal classification results; however, in this research, all NIR spectra were preprocessed by moving the average and standard normal variate (SNV) for the purposes of comparison [20].

#### 3.3.3. Davies–Bouldin Index (DBI)

To evaluate whether the clusters of the samples could be well-separated after the transformation process, the Davies–Bouldin index (DBI) was employed [28,30]. The DBI is a commonly used cluster validation index that makes comparisons between inter-cluster and intra-cluster distances of the sample clusters. In this research, the inter-cluster distance was the Euclidean distance between the centroids of the inspecting clusters, and the intra-cluster distance referred to the Euclidean distance between the two most-distanced samples from each cluster. A smaller value of DBI indicates a greater degree of separation between the clusters. The DBI values reported in this research were the summation of the DBI values among the three class memberships of the coffee samples.

#### 3.3.4. Model Statistics and Validation

**Figure 7:**
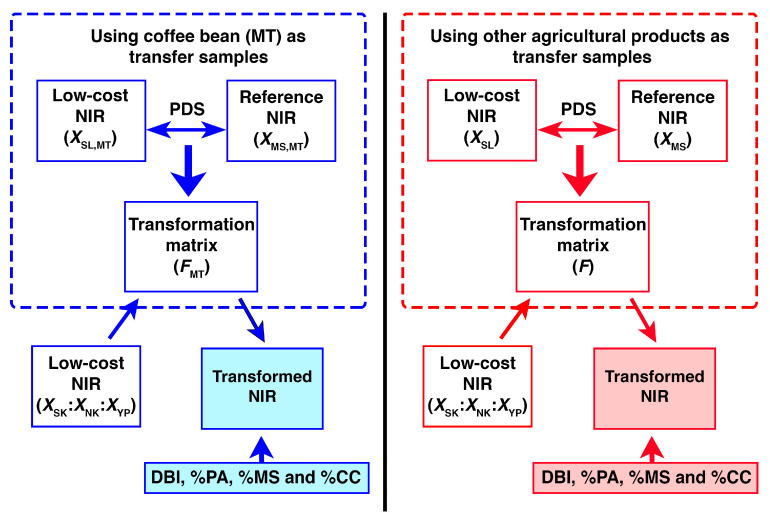
Overview of the experimental procedure of this research. Transformations using green coffee beans (MT) (left) and various agricultural samples (right). ***X***_SL,MT_ and ***X***_MS,MT_ represent the NIR spectra of the coffee samples (MT), respectively, which were used as the slave and master data for the PDS transformation. ***X***_SL_ and ***X***_MS_ represent the NIR spectra of the other agricultural samples obtained from the homemade and NIRSystem 6500 spectrometers. ***X***_SK_:***X***_YP_:***X***_NK_ represent the NIR spectra of the coffee samples (SK, YP, and NK) recorded using the homemade instrument.

The characteristics of the classification models could be dependent upon the training samples used to establish the models. Different selections of the training samples, in many cases, could result in different optimal solutions for the same problem. In this research, the predictive performance of the developed models was evaluated using a bootstrap methodology [28]. In this case, 2/3 of the samples acquired from each class membership were randomly selected and used as training samples, while the rest of the samples were used as test samples. This algorithm was repeated 50 times. After that, some statistical indices based on a majority vote, including the percentages of predictive ability (% PA), model stability (% MS), and correctly classified instances (% CC) were calculated to evaluate the accuracy, stability, and performance of the classification models [31,32]. A diagram of the experimental procedure employed in this research is presented in Figure 7. Calculations of PDS, SSOM, DBI, and the model statistics were achieved using in-house MATLAB scripts (MATLAB V10.0, The Math Works Inc., Natick, MA, USA).

## 4. Conclusions

The developed NIR spectrometer could be applied to classifying green coffee beans based on their geographical origins using SSOM. Spectral transformation could improve the classification performance of the homemade NIR spectrometer by incorporating a fraction of the information in the NIR spectra acquired from the reference NIR instrument. A variety of the transfer samples could then be used for the transformation process. Moreover, it was not necessary that the transfer samples share the same characteristics as the test samples. The suitability of the transfer samples could be evaluated using the DBI cluster validation index. In this demonstration, the transformation of the green coffee spectra, based on the use of corn as the transfer sample, could improve the classification performance by up to 23% in terms of classification accuracy when compared to the classification performance of the non-transformation data. Therefore, the developed method could improve the accuracy and reliability of the developed NIR instrument without additional costs, based on the previously provided database of the agricultural transfer samples.

## Figures and Tables

**Table 1:** Comparison of classification performance.

Spectrometer	Transfer Sample	% PA		% MS		% CC		DBI	−log(DBI)
		Tr *	Ts **	Tr	Ts	Tr	Ts		
NIRSystem 6500	-	100	98	100	96	100	100	0.39	0.94
Homemade instrument	-	98	61	96	58	100	64	5.75	−1.75
	Corn	97	71	95	67	100	79	3.03	−1.11
	Red bean	96	71	93	67	99	75	4.09	−1.41
	Mung bean	97	59	93	60	100	67	2.77	−1.02
	White rice	96	66	92	58	99	75	3.53	−1.26
	Green coffee (MT)	99	70	99	65	100	78	2.49	−0.91
	Black bean	96	59	92	66	100	61	4.83	−1.57
	Roasted coffee	98	55	96	56	100	61	4.31	−1.46
	Adzuki bean	93	46	87	53	100	52	12.72	−2.54
	Paddy rice	92	51	85	52	99	52	6.38	−1.85
	Soybean	91	52	82	57	99	59	13.36	−2.59

* Tr = Prediction for the training samples. ** Ts = Prediction for the test samples.

**Table 2:** Details of the green coffee samples.

Plantation Area	No. of Samples	Altitude (m)	Width (mm)	Length (mm)	Thickness (mm)	Weight (g)	Density (g/cm^3^)
Sop Khong (SK)	36	1107	7.6 ± 0.6	10.7 ± 0.9	4.6 ± 0.6	0.137 ± 0.03	0.99 ± 0.22
Yang Piang (YP)	27	936	8.0 ± 0.6	10.4 ± 1.1	4.9 ± 0.5	0.152 ± 0.03	1.01 ± 0.24
Mae Tuen (MT)	24	441	8.1 ± 0.5	11.1 ± 1.1	4.9 ± 0.5	0.156 ± 0.03	0.95 ± 0.14
Na Kian (NK)	24	1212	7.9 ± 0.6	10.5 ± 0.9	4.9 ± 0.4	0.142 ± 0.03	0.92 ± 0.12

Values represent mean ± standard deviation of 60 beans.

## Data Availability

The datasets and codes are available upon request.

## References

[1] Giraudo A., Grassi S., Savorani F., Gavoci G., Casiraghi E., Geobaldo F. (2019). Determination of the geographical origin of green coffee beans using NIR spectroscopy and multivariate data analysis. Food Control.

[2] Luykx D.M., van Ruth S.M. (2008). An overview of analytical methods for determining the geographical origin of food products. Food Chem..

[3] Cortés V., Blasco J., Aleixos N., Cubero S., Talens P. (2019). Monitoring strategies for quality control of agricultural products using visible and near-infrared spectroscopy: A review. Trends Food Sci. Technol..

[4] Lin M., Mousavi M., Al-Holy M., Cavinato A.G., Rasco B.A. (2006). Rapid Near Infrared Spectroscopic Method for the Detection of Spoilage in Rainbow Trout (Oncorhynchus mykiss) Fillet. J. Food Sci..

[5] Farres S., Srata L., Fethi F., Kadaoui A. (2019). Argan oil authentication using visible/near infrared spectroscopy combined to chemometrics tools. Vib. Spectrosc..

[6] Hespanhol M.C., Pasquini C., Maldaner A.O. (2019). Evaluation of a low-cost portable near-infrared spectrophotometer for in situ cocaine profiling. Talanta.

[7] Modroño S., Soldado A., Martínez-Fernández A., de la Roza-Delgado B. (2017). Handheld NIRS sensors for routine compound feed quality control: Real time analysis and field monitoring. Talanta.

[8] Cruz-Tirado J.P., da Silva Medeiros M., Barbin D.F. (2021). On-line monitoring of egg freshness using a portable NIR spectrometer in tandem with machine learning. J. Food Eng..

[9] Sedjoah R.-C.A.-A., Ma Y., Xiong M., Yan H. (2021). Fast monitoring total acids and total polyphenol contents in fermentation broth of mulberry vinegar using MEMS and optical fiber near-infrared spectrometers. Spectrochim. Acta Part A Mol. Biomol. Spectrosc..

[10] Sharififar A., Singh K., Jones E., Ginting F.I., Minasny B. (2019). Evaluating a low-cost portable NIR spectrometer for the prediction of soil organic and total carbon using different calibration models. Soil Use Manag..

[11] van Kollenburg G.H., van Manen H.-J., Admiraal N., Gerretzen J., Jansen J.J. (2021). Low-cost handheld NIR spectroscopy for identification of organic solvents and low-level quantification of water contamination. Talanta.

[12] Workman J.J. (2018). A Review of Calibration Transfer Practices and Instrument Differences in Spectroscopy. Appl. Spectrosc..

[13] Hernández N., Gracia A., León L., Barreiro P., Herrero D. (2008). Calibration transfer between portable and laboratory NIR spectrophotometers. Acta Hortic..

[14] da Silva N.C., Cavalcanti C.J., Honorato F.A., Amigo J.M., Pimentel M.F. (2017). Standardization from a benchtop to a handheld NIR spectrometer using mathematically mixed NIR spectra to determine fuel quality parameters. Anal. Chim. Acta.

[15] Milanez K.D.T.M., Silva A.C., Paz J.E.M., Medeiros E.P., Pontes M.J.C. (2016). Standardization of NIR data to identify adulteration in ethanol fuel. Microchem. J..

[16] Abdelkader M.F., Cooper J.B., Larkin C.M. (2012). Calibration transfer of partial least squares jet fuel property models using a segmented virtual standards slope-bias correction method. Chemom. Intell. Lab. Syst..

[17] Pissard A., Marques E.J.N., Dardenne P., Lateur M., Pasquini C., Pimentel M.F., Pierna J.A.F., Baeten V. (2021). Evaluation of a handheld ultra-compact NIR spectrometer for rapid and non-destructive determination of apple fruit quality. Postharvest Biol. Technol..

[18] Gonjo T., Futami Y., Morisawa Y., Wojcik M.J., Ozaki Y. (2011). Hydrogen Bonding Effects on the Wavenumbers and Absorption Intensities of the OH Fundamental and the First, Second, and Third Overtones of Phenol and 2,6-Dihalogenated Phenols Studied by Visible/Near-Infrared/Infrared Spectroscopy. J. Phys. Chem. A.

[19] Krongchai C., Funsueb S., Jakmunee J., Kittiwachana S. (2017). Application of multiple self-organizing maps for classification of soil samples in Thailand according to their geographic origins. J. Chemom..

[20] Phuangsaijai N., Theanjumpol P., Muenmanee N., Kittiwachana S. (2021). Fabrication of a low-cost NIR spectrometer for detection of agricultural product quality. Chiang Mai J. Sci..

[21] Kaewpangchan P., Phuangsaijai N., Seehanam P., Theanjumpol P., Maniwara P., Kittiwachana S. (2021). Screening of coffee impurity using a homemade NIR sensor system. Chiang Mai J. Sci..

[22] Ji W., Viscarra Rossel R.A., Shi Z. (2015). Improved estimates of organic carbon using proximally sensed vis-NIR spectra corrected by piecewise direct standardization. Eur. J. Soil Sci..

[23] Brereton R.G. (2003). Chemometrics: Data Analysis for the Laboratory and Chemical Plant.

[24] Bouveresse E., Massart D. (1996). Improvement of the piecewise direct standardisation procedure for the transfer of NIR spectra for multivariate calibration. Chemom. Intell. Lab. Syst..

[25] Wongsaipun S., Theanjumpol P., Kittiwachana S. (2021). Development of a Universal Calibration Model for Quantification of Adulteration in Thai Jasmine Rice Using Near-infrared Spectroscopy. Food Anal. Methods.

[26] Astel A., Tsakovski S., Barbieri P., Simeonov V. (2007). Comparison of self-organizing maps classification approach with cluster and principal components analysis for large environmental data sets. Water Res..

[27] Kittiwachana S., Wangkarn S., Grudpan K., Brereton R.G. (2013). Prediction of liquid chromatographic retention behavior based on quantum chemical parameters using supervised self organizing maps. Talanta.

[28] Brereton R.G. (2009). Chemometrics for Pattern Recognition.

[29] Luna A.S., da Silva A.P., Alves E.A., Rocha R.B., Lima I.C.A., de Gois J.S. (2017). Evaluation of chemometric methodologies for the classification of Coffea canephora cultivars via FT-NIR spectroscopy and direct sample analysis. Anal. Methods.

[30] Dixon S.J., Heinrich N., Holmboe M., Schaefer M.L., Reed R.R., Trevejo J., Brereton R.G. (2009). Use of cluster separation indices and the influence of outliers: Application of two new separation indices, the modified silhouette index and the overlap coefficient to simulated data and mouse urine metabolomic profiles. J. Chemom..

[31] Theanjumpol P., Wongzeewasakun K., Muenmanee N., Wongsaipun S., Krongchai C., Changrue V., Boonyakiat D., Kittiwachana S. (2019). Non-destructive identification and estimation of granulation in ‘Sai Num Pung’ tangerine fruit using near infrared spectroscopy and chemometrics. Postharvest Biol. Technol..

[32] Dixon S.J., Xu Y., Brereton R.G., Soini H.A., Novotny M.V., Oberzaucher E., Grammer K., Penn D.J. (2007). Pattern recognition of gas chromatography mass spectrometry of human volatiles in sweat to distinguish the sex of subjects and determine potential discriminatory marker peaks. Chemom. Intell. Lab. Syst..

